# Genome-Wide Analyses of Prognostic and Therapeutic Alternative Splicing Signatures in Bladder Urothelial Carcinoma

**DOI:** 10.3389/fonc.2021.626858

**Published:** 2021-03-26

**Authors:** Zhongru Fan, Zhe Zhang, Chiyuan Piao, Zhuona Liu, Zeshu Wang, Chuize Kong

**Affiliations:** Department of Urology, The First Affiliated Hospital of China Medical University, China Medical University, Shenyang, China

**Keywords:** alternative splicing (AS), bladder urothelial carcinoma (BLCA), prognosis signature, regulatory network, splicing factor, immuno/chemotherapies

## Abstract

**Background:**

Alternative splicing (AS) is an indispensable post-transcriptional modification applied during the maturation of mRNA, and AS defects have been associated with many cancers. This study was designed to thoroughly analyze AS events in bladder urothelial carcinoma (BLCA) at the genome-wide level.

**Methods:**

We adopted a gap analysis to screen for significant differential AS events (DASEs) associated with BLCA. DASEs with prognostic value for OS and the disease-free interval (DFI) were identified by Cox analysis. In addition, a differential AS network and AS clusters were identified using unsupervised cluster analysis. We examined differences in the sensitivity to chemotherapy and immunotherapy between BLCA patients with high and low overall survival (OS) risk.

**Results:**

An extensive number of DASEs (296) were found to be clinically relevant in BLCA. A prognosis model was established based prognostic value of OS and DFI. CUGBP elav-like family member 2 (CELF2) was identified as a hub splicing factor for AS networks. We also identified AS clusters associated with OS using unsupervised cluster analysis, and we predicted that the effects of cisplatin and gemcitabine chemotherapy would be different between high- and low-risk groups based on OS prognosis.

**Conclusion:**

We completed a comprehensive analysis of AS events in BLCA at the genome-wide level. The present findings revealed that DASEs and splicing factors tended to impact BLCA patient survival and sensitivity to chemotherapy drugs, which may provide novel prospects for BLCA therapies.

## Introduction

Bladder urothelial carcinoma (BLCA) is a common genitourinary malignancy, with an estimated 430,000 cases diagnosed annually worldwide, associated with 165,000 deaths (1). Some effective methods used for diagnosis and treatment include intravesical Bacillus Calmette and Guérin, which is used to treat intermediate- and high-risk, non-muscle-invasive bladder cancer; and immunotherapy with checkpoint inhibition, targeted therapies, and antibody–drug conjugates, which are used to treat muscle-invasive and advanced diseases. These treatments have been developed due to the profound understanding of the molecular biology and genetics underlying BLCA (2). However, studies are continuously necessary to continue probing unexploited mechanisms for the treatment of BLCA. One study identified over 4,632 survival-associated alternative splicing (AS) events (SASEs) in BLCA and indicated that the overall incidence of SASEs correlated strongly with survival (3), which indicated that AS might be a noteworthy regulatory mechanism in BLCA.

The AS process represents a critical post-transcriptional modification that allows for a single gene to produce diverse mRNA and protein isoforms, contributing to the rich proteome in somatic cells (4). Aberrations in splicing events and their regulators, which are known as splicing factors (SFs), can lead to the development and progression of cancer (5). The identified correlations between AS and some cancers, such as prostate, lung, gastric, and breast cancers, have suggested that AS may serve as a cancer hallmark and treatment target (6–9). Researchers have long recognized that AS events are relevant to bladder cancer (10). Recently, studies have expanded the exploration of the SF–AS regulatory pathway in tumor biology and function in BLCA. For example, polypyrimidine tract-binding protein 1 (PTBP1) directly regulates the splicing of pyruvate kinase isozyme M2 (PKM2) and MEIS2-L, and these two splicing events induce cell proliferation and lymph node metastasis, respectively (11). Similarly, non-POU domain-containing octamer-binding protein (NONO) can mediate a series of oncogenic expression events by regulating the SET domain and mariner transposase fusion gene (SETMAR) (12). The AS–SF network appears to play a strong regulatory role in BLCA. Therefore, the in-depth analysis of AS in BLCA at the whole-genome level may be clinically relevant.

Bioinformatics analyses examining AS in recent years have commonly been based on SASEs, which has allowed for the construction of prognostic models with good performance. To determine intrinsic discrepancies between tumor and normal tissues, gap analysis is crucial for oncology research. Differential AS events (DASEs) describe discrepancies in the splice sites between a pair of samples, which is vital to understanding AS and its regulatory mechanisms. Thus, we aimed to explore DASEs in BLCA.

In this study, we systematically analyzed DASEs using data obtained from The Cancer Genome Atlas (TCGA) SpliceSeq database and prognosis biomarkers associated with BLCA. We conducted survival analyses and established an overall survival (OS) and DFI prognosis model for BLCA. Based on our results, we explored differences in the sensitivity to immunotherapy and chemotherapy among BLCA patients with high or low OS risk. In addition, we performed an unsupervised cluster analysis and constructed a differential AS network, in which we defined three sample clusters and identified eight key SFs associated with 186 DASEs.

## Materials and Methods

### Data Gathering and Processing

**Figure 1:**
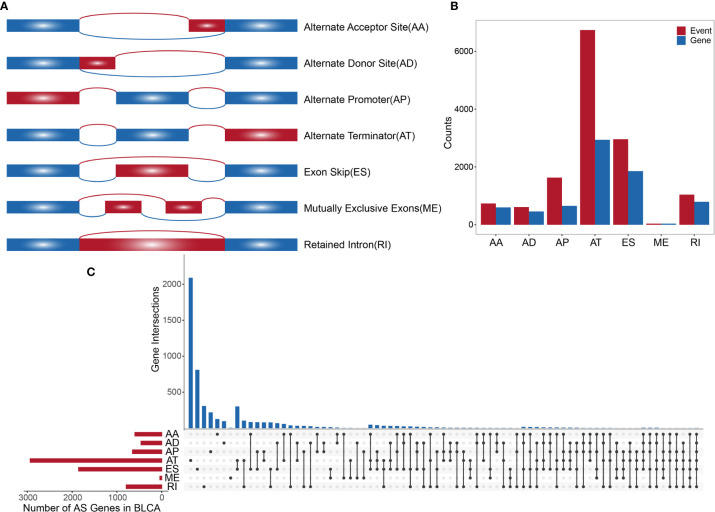
**(A)** Schematic diagram of AS. **(B)** Histogram of overall AS events and the number of genes involved. **(C)** The UpSetR plot showing the relationships between overall AS event-related genes across different types.

TCGA SpliceSeq (https://bioinformatics.mdanderson.org/TCGASpliceSeq/) is a database for studying the splicing patterns identified among TCGA RNA sequencing (RNAseq) data. The percent spliced in (PSI) value, which is an intuitive ratio ranging from 1 to 0, can be utilized to quantify AS events and categorize seven AS types: alternate acceptor site (AA), alternate donor site (AD), alternate promoter (AP), alternate terminator (AT), exon skip (ES), mutually exclusive exons (ME), and retained intron (RI) (**Figure 1A**) (13). Following the standards of “the percentage of samples with PSI = 100%”, we screened the splicing patterns of protein-encoding genes among BLCA patients. The upsetR package was used to draw an upsetR plot to describe the quantity of genes alternatively spliced. We also obtained RNAseq data for BLCA patients from TCGA (using the Genomic Data Commons data portal at https://portal.gdc.cancer.gov/). Clinical data, including survival, age, sex, and cancer stage, were obtained from UCSC Xena (http://xena.ucsc.edu/). The inclusion criteria for BLCA patient samples included date regarding survival time and survival state and OS > 30 days. We included 425 cancer-related samples (including 406 tumor tissues and 19 normal adjacent tissues) in our study, based on the integration of AS data, expression profiles, and other clinical information (**Table 1**). All statistical analysis in the context were performed using R (version: 3.6.2).

**Table 1:** Clinical features of bladder urothelial carcinoma.

Clinical Features		Patient	Percent (%)
OS	Alive	229	56.40
	Dead	177	43.60
OS Time	≤ 5 years	358	88.18
	> 5 years	47	11.58
	Missing Value	1	0.24
DFI	Disease-Free	155	38.18
	Recurrence	31	7.64
	Missing Value	220	54.18
DFI time	≤ 5 years	160	39.41
	> 5 years	26	6.40
	Missing Value	220	54.19
Sex	Female	105	25.86
	Male	301	74.14
Age	≤ 60	107	26.35
	> 60	299	73.65
T	T0	1	0.24
	T1	3	0.74
	T2	119	29.31
	T3	192	47.29
	T4	58	14.29
	Missing Value	33	8.13
N	N0	235	57.88
	N1	46	11.33
	N2	75	18.47
	N3	8	1.97
	Missing Value	42	10.35
M	M0	196	48.28
	M1	11	2.71
	Missing Value	199	49.01
Stage	I	2	0.49
	II	130	32.02
	III	138	33.99
	IV	134	33.01
	Missing Value	2	0.49
**Total**		**406**	**100**

OS, overall survival; DFI, disease-free survival; T, tumor; N, node; M, metastasis.

### Differential Splicing Event Analysis

We compared tumor samples with adjacent normal tissue samples to identify DASEs with an average PSI > 0.05. The Wilcoxon rank-sum test was performed to evaluate the significance of DASEs between samples, and the Benjamini–Hochberg method was used to correct for multiple testing. We then defined DASEs with adjusted P-values < 0.05 and |log_2_ (fold change)| > 1 as significant. To detect commonly occurring AS events, the following quality control rules were defined: first, the percentage of samples with PSI = 100% were included, and, second, the average PSI > 0.05. This allowed for the exclusion of rare AS events. We used pheatmap R package to draw a heatmap of top 20 DASEs and ggpubr package to draw a box plot of top 3 DASEs in order to show overall condition of DASEs in BLCA. Therefore, the model established here can be applied to non-special and larger sample populations. In addition, we also analyzed the differential expression of protein-encoding genes between tumor tissues and normal adjacent tissues using the edgeR package (standardized by calcNormFactors [expr, method = “TMM”] in edgeR). Differentially expressed genes (DEGs) were corrected by the Benjamini–Hochberg method by defining significant DEGs as those with P-values < 0.05 and |log_2_ (fold change)| > 1. To further understand the regulatory role played by AS-associated genes in BLCA, we submitted the identified DASE-related genes to the STRING database (www.string-db.org/) to generate a protein–protein interaction (PPI) network. The “multiple proteins” column was selected.

### Survival Analysis

First, we used a survival R package to perform a univariate Cox regression analysis to identify correlations between DASEs and survival in BLCA (including OS and DFI; samples with OS and DFI values greater than 30 days were retained for analysis). Second, the top 10 survival-related DASEs in BLCA were included in the stepwise Cox regression analysis, and a prognostic risk score was determined based on a linear combination of the AS PSI multiplied by the corresponding regression coefficient (b), which was used to represent the correlation weight. This regression coefficient was calculated from the multivariate Cox proportional hazard regression model, and the risk score formula was as follows:

$$Risk\;Score=PSI\;of\;AS_{1}\;\times \;b_{AS1}+PSI\;of\;AS_{2}\;\times b_{AS2}+\ldots +\;PSI\;of\;AS_{10}\;\times b_{AS10}$$

$$Risk\;Score_{OS}=PSI\;of\;AS_{3412\_PTGER3\_AT}\;\times \;1.937+PSI\;of\;AS_{46432\_CIRBP\_RI}\;\times -1.890+PSI\;of\;AS_{30219\_CCNDBP1\_AA}\;\times -3.301$$

$$\begin{array}{c} \mathrm{Risk}\;\mathrm{Score}{}_{DFI}=PSI\;of\;AS_{84100\_C8orf34\_AT}\times -5.738+PSI\;of\;AS_{82597\_TNKS\_AT}\times -6.495\\ +PSI\;of\;AS_{63304\_FANCD2\_AT}\times 6.383+PSI\;of\;AS_{48124\_TPM4\_AP}\times 1.028\\ +PSI\;of\;AS_{84681\_COX6C\_AT}\times -2.798 \end{array}$$

Based on the results of the stepwise Cox regression analysis, prognostic AS events in BLCA were identified, and corresponding OS and DFI prognostic models were constructed. We used the survminer R package to draw a Kaplan–Meier curve, which shows the top 10 individual DASEs and survival times to determine whether the prognosis models were able to distinguish favorable or poor patient prognoses. We calculated the area under the receiver operating characteristic (ROC) curve (AUC) using a survivalROC R package to further evaluate the OS and DFI prognosis models over a 5-year survival period.

### The Construction of an Alternative Splicing Network

The SF is a key regulator of AS. In the tumor microenvironment, a limited number of SFs can regulate multiple AS events. First, we collated a list of human SFs from a human SF database (14, 15). Second, we extracted SF-related gene expression profile data for BLCA, analyzed the identified SFs with an edgeR package, and corrected them using the Benjamin–Hochberg method. SFs with P-values < 0.05 and |log_2_ (fold change)| > 1 were defined as differential expressing SFs. Third, the Spearman test was used to analyze the potential regulatory correlations between the expression of various SFs and the occurrence of DASEs, in which correlations with P-values < 0.05 and |R| > 0.4 were deemed significant. The regulatory network of AS events and SFs in BLCA was constructed by using Cytoscape (version:3.6.0). Finally, we adopted the ClueGO plug-in for Cytoscape to analyze the gene ontology (GO) and functional enrichment of the related genes in the network, and we identified significantly related GO terms (P-value < 0.05). In addition, univariate Cox regression analysis and survival analysis were employed to identify the impacts of identified SFs on survival.

### Identification of Alternative Splicing Clusters Associated With Prognosis and Molecular Subtypes

AS events vary greatly at the individual level. We applied an unsupervised consensus method performed by ConsensusClusterPlus R package to identify AS clusters for BLCA (related parameters: distance = “Euclidean”; clusterAlg = “km”). We analyzed the relationships between AS clusters and survival time and further examined relevant clinical information (including age, sex, T, N, M, and stage) to identify associations between clinical information and AS clusters.

### Predictions for Immunotherapy and Chemotherapy

**Figure 2:**
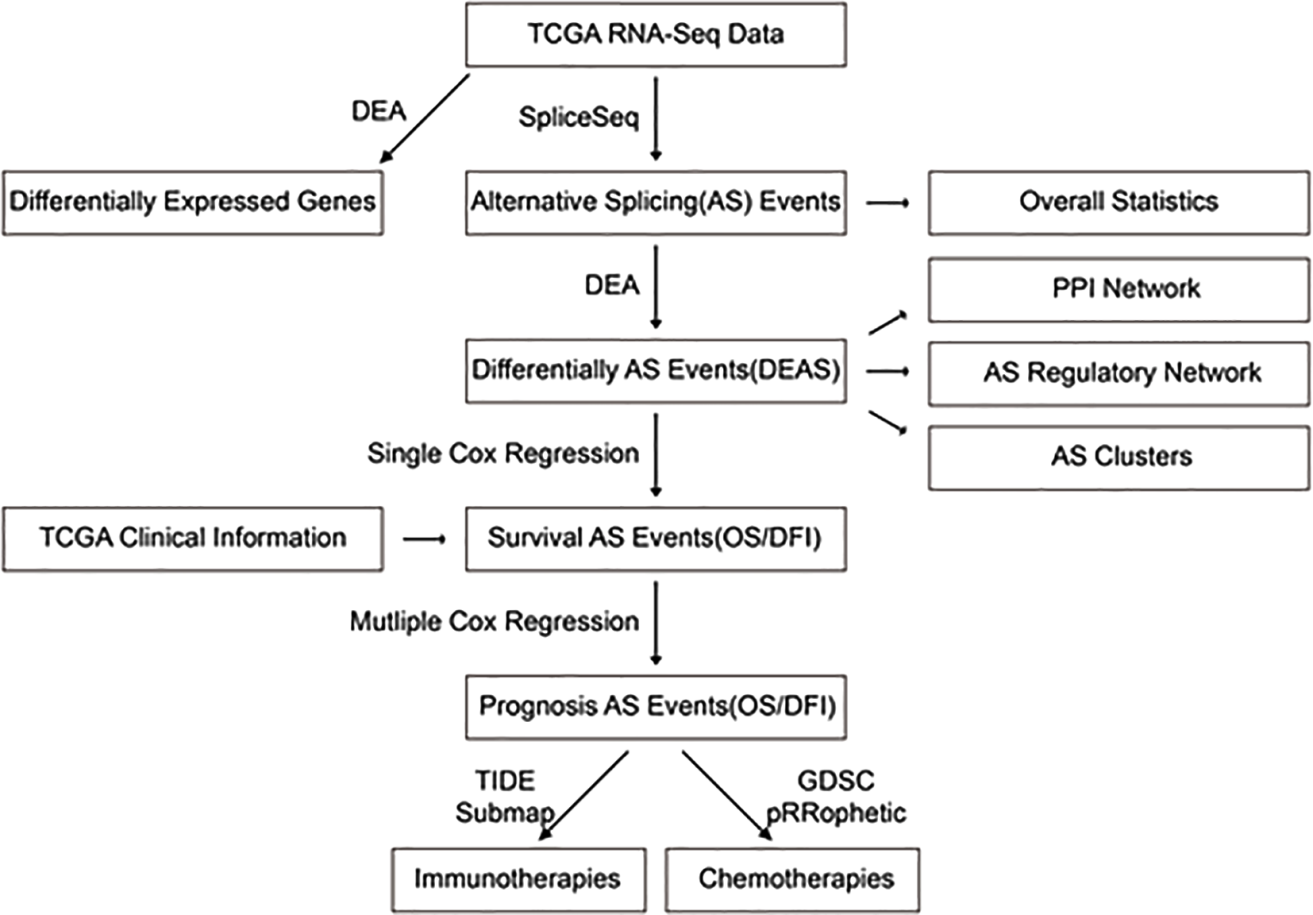
Overall framework of the study.

Based on the data obtained from the publicly available pharmacogenomics database, The Genomics of Drug Sensitivity in Cancer (GDSC at https://www.cancerrxgene.org/) (16), we predicted the chemotherapeutic response of each sample. During this process, the pRRophetic R package was used to generate forecasts, in which the minimal inhibitory concentration (IC_50_) value of the sample was estimated by ridge regression, and the prediction accuracy was evaluated based on a ten-fold cross-validation of the GDSC training set (pRRopheticPredict [test matrix = Data; drug = Drug; tissue type = “allSolidTumors”; batchCorrect = “eb”; remove Low Varying Genes = 0.2], all other parameters were set to default). We selected two commonly used chemicals (cisplatin and gemcitabine) to individually predict the IC_50_ values of each BLCA sample, and we calculated the differences in chemotherapeutic responses between the two drugs for the high- and low-risk groups, categorized by the AS-based OS prognosis using the Wilcoxon rank-sum test (P-values < 0.05). We also utilized the submap algorithm of TIDE (http://tide.dfci.harvard.edu/) and GenePattern (https://cloud.genepattern.org/gp) to predict discrepancies in the clinical responses to immune checkpoint blockades among BLCA patients who were at either high or low risk, according to the AS-based OS prognoses. On the TIDE, we chose “others” in the column “Cancer type” and “no” in the column of “Previous immunotherapy.” Fisher’s exact test was used to verify the relevance between OS-grouping and the immunotherapy response. On GenePattern, a submap was used for analysis and Bonferroni’s *post hoc* test was used to correct P-values. The overall framework of this study is shown in **Figure 2**.

## Results

### Overview of Alternative Splicing Events in BLCA

A synthetic analysis of AS profiles in human BLCA was employed. A total of 13,747 AS events associated with 5,174 genes were identified. In detail, we detected 736 instances of the AA splice type, involving 598 genes; 609 instances of the AD splice type, involving 459 genes; 1,629 instances of the AP splice type, involving 651 genes; 6,739 instances of the AT splice type, involving 2,937 genes; 2,957 instances of the ES splice type, involving 1,855 genes; 38 instances of the ME splice type, involving 38 genes; and 1,039 instances of the RI splice type, involving 791 genes, as shown in **Figures 1A, B**. The AT splice type was the most common type identified (> 49%), and ES was the second most frequent type (> 21%), whereas ME was the rarest type. A given gene could be associated with multiple types of AS events, with some genes associated with up to five or six variable splicing types (**Figure 1C**). The information of 425 included samples is shown in **Supplementary Table S1**.

### Identification of Differential Alternative Splicing Events

**Figure 3:**
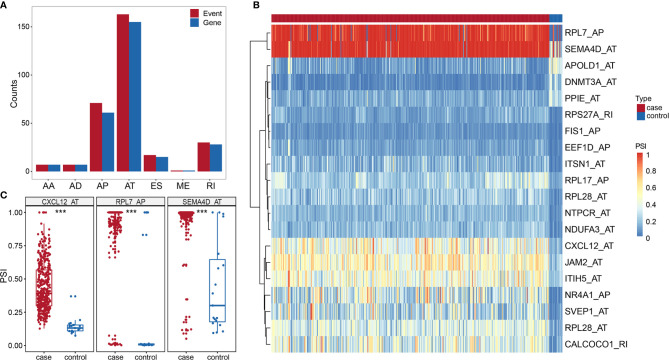
**(A)** Histogram of DASE distribution in BLCA. **(B)** Heat map of the top 20 differentially AS events in BLCA. **(C)** Box plot of variable shear events among the top three alternative splice events in BLCA. *** represents p values < 0.0001.

We identified 296 DASEs by comparing the BLCA group with the control group, associated with 272 genes (**Figure 3A**). To investigate the relationship between DEGs and DASEs, 4,752 DEGs were identified in BLCA compared with the control group (2,679 upregulated genes and 2,073 downregulated genes) Representative DASE are shown as heat plot (**Figure 3B**) and box plot (**Figure 3C**).The results of all and selected DASEs and DEGs were offered as **Supplementary Table S2**-**S5**.

### The Construction of the PPI Network

**Figure 4:**
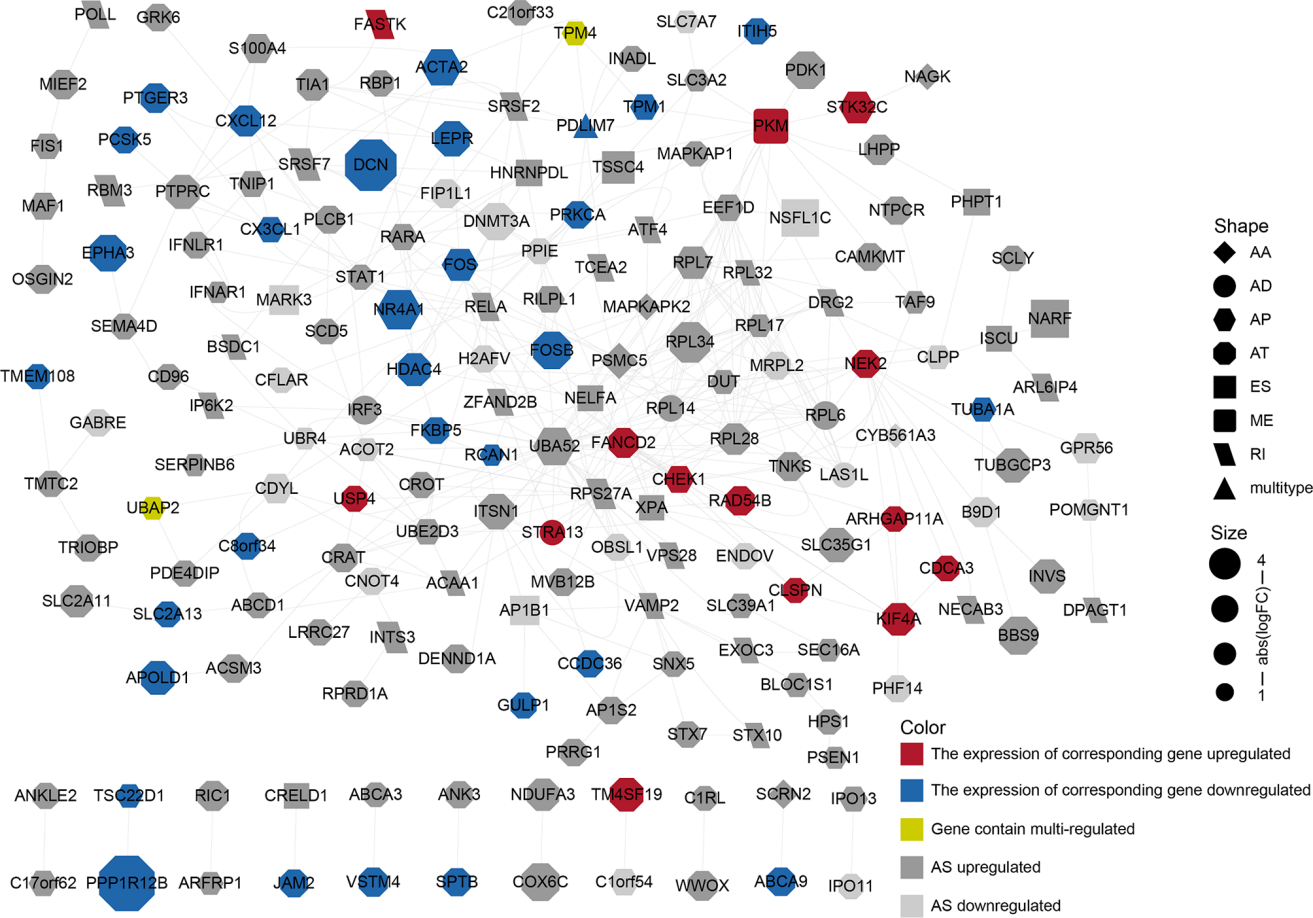
PPI network constructed by different alternative splicing-related genes in BLCA. The dots represent alternatively spliced genes, whereas the edges represent the relationships between the proteins corresponding to those genes. The shapes of the dots represent the AS types; the color of the dots represents the changes in gene expression. The size of the node represents |log2 (fold change)|.

We performed a PPI network analysis of differentially AS-related genes in BLCA and identified several hub genes based on the number of collected genes. We identified 186 nodes and 392 edges in the PPI network, including the hub nodes *UBA52* (degree = 35), *RPS27A* (degree = 32), *PSMC5* (degree = 16), *RPL7* (degree = 15), and *PKM* (degree = 15) (**Figure 4**). The GO analysis of proteins in the network was shown in **Supplementary Table S6**.

### The Construction of a Prognostic Alternative Splicing Event Model

**Figure 5:**
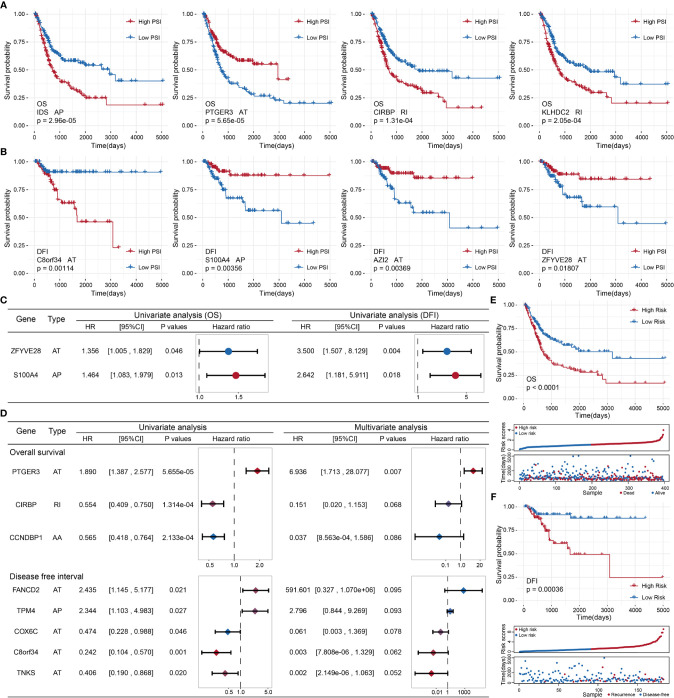
**(A)** Kaplan–Meier curve of the four AS events associated with OS. **(B)** Kaplan–Meier curve of the top four AS events associated with DFI. **(C)** Comparison of AS events associated with OS and DFI in univariate Cox regression analysis of BLCA. **(D)** Comparison of univariate Cox analysis and stepwise Cox analysis of AS events associated with OS and DFI prognoses. **(E, F)**: Kaplan–Meier plot, risk score plot, and survival state plot of OS and DFI prognostic models for BLCA.

To probe the prognostic value of AS events in BLCA patients, we first adopted a univariate Cox regression analysis to evaluate the influence of AS events on the prognoses of BLCA patients. We detected 87 OS-related and 12 DFI-related AS events among the identified DASEs in BLCA. Both groups of AS events were most commonly associated with the AT and AP types (21 APs and 33 ATs in the OS group, accounting for > 62%; all DFIs were either AP or AT types, with 3 APs and 9 ATs). We also identified events that were related to both OS and DFI (total two), and plotted a forest map (**Figure 5C**).

Next, we attempted to identify independent prognostic factors associated with BLCA patients. We selected the top 10 OS- and DFI-related AS events in BLCA as candidate factors and utilized a stepwise Cox regression analysis to select independent prognostic-related AS events to establish various prognostic models (the top four event-related survival curves are shown in **Figures 5A, B**; the remaining six curves are shown in **Supplementary Figure S1**). Three independent prognostic factors were associated with OS, and five independent prognostic factors were associated with DFI (**Figure 5D**). In the light of the median risk scores calculated for the OS and DFI prognostic models, BLCA patients were separated into a low-risk group and a high-risk group. Both the OS and DFI prognostic models showed the significant ability to differentiate survival among BLCA patients, and the DFI model showed better performance (OS: p = 1.03505e−05, AUC = 0.6767398; DFI: p = 0.0003621185, AUC = 0.8965976; see **Figures 5E, F**). The detailed parameters of clusters are submitted as “**Data Sheet File** for clustering”.

### The Construction of an Alternative Splicing Network Based on Gap Analysis

**Figure 6:**
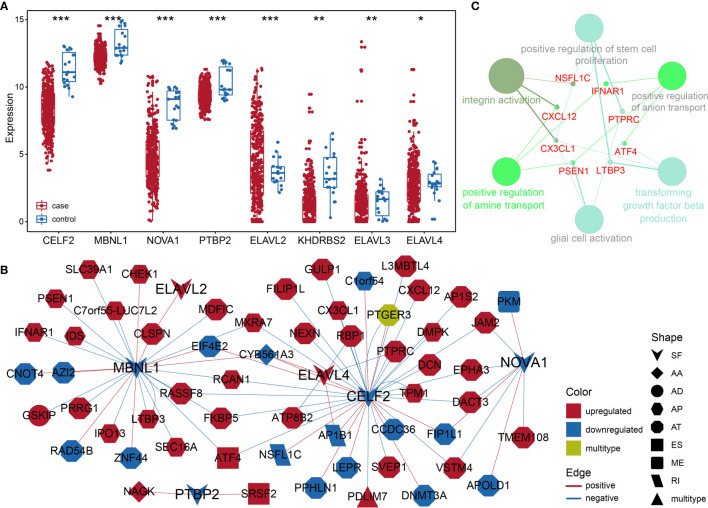
**(A)** Box plot of differentially expressed SFs in BLCA. **(B)** Different AS networks in BLCA. **(C)** Gene-rich GO terms in different AS networks in BLCA. * represents p values< 0.05, ** represents p values < 0.01, *** represents p values< 0.001.

**Figure 7:**
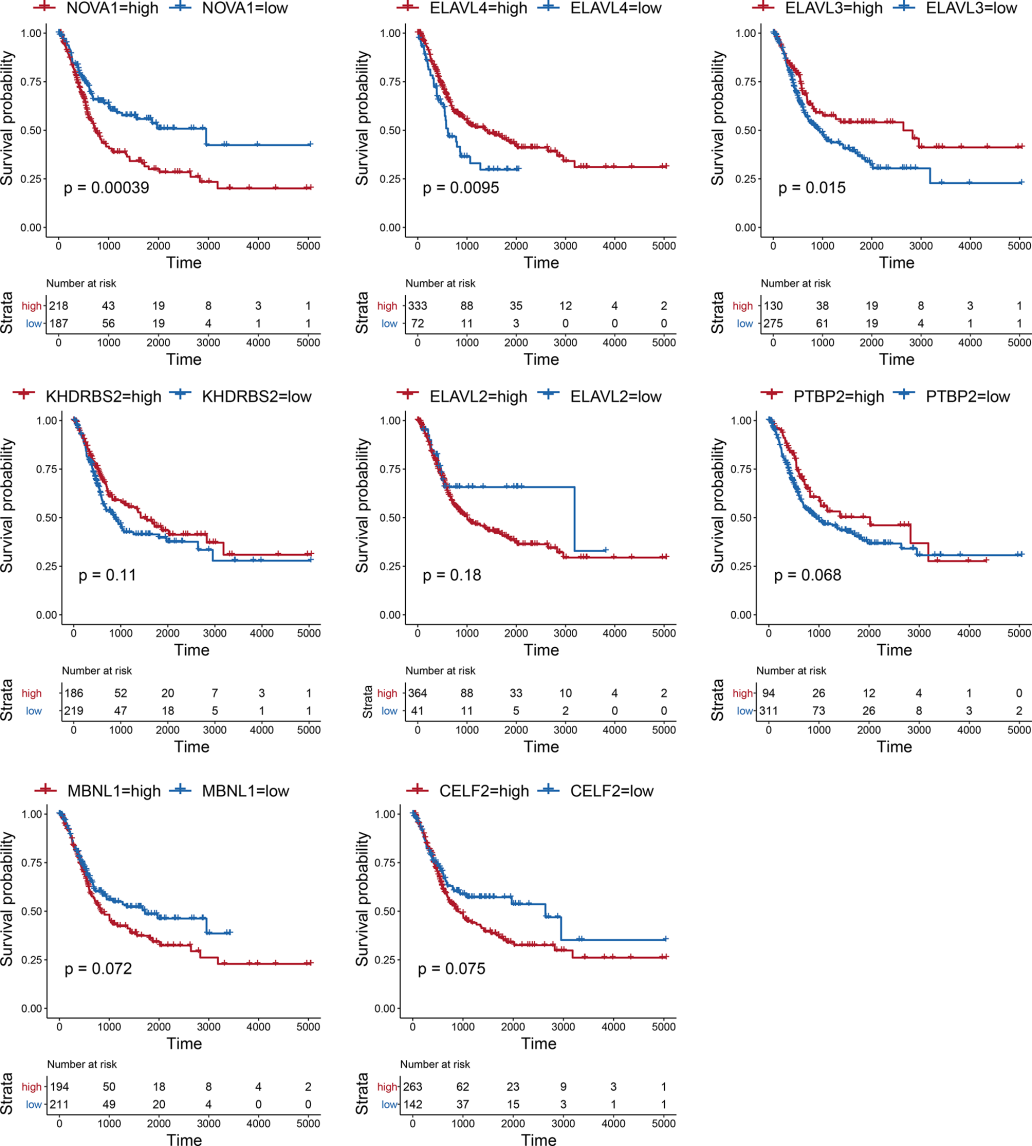
Kaplan–Meier survival curve for eight hub SFs.

Considering the notable differences in AS events in BLCA, we further analyzed the relationships between AS events and SFs. First, we investigated the differentially expressed SFs in BLCA, and we distinguished eight differential SFs: *CELF2*, *MBNL1*, *NOVA1*, *PTBP2*, *KHDRBS2*, *ELAVL2*, *ELAVL3*, and *ELAVL4*. Of these, *ELAV2*, *ELAVL3*, and *ELAVL4* were upregulated in BLCA, and *CELF2*, *MBNL1*, *NOVA1*, *PTBP2*, and *KHDRBS2* were downregulated (**Figure 6A**). Then we evaluated the correlations between DASEs and differentially expressed SFs, and we chose highly correlated pairs (|R| > 0.4 and P-value < 0.05) to generate a differential AS network. Among these SFs, *CELF2* is a pivotal splicing factor in the network, associated with 37 different AS events but is also negatively correlated with 26 different AS events. The *MBNL1* and *NOVA1* SFs also tended to be negatively correlated with most AS events (**Figure 6B**). In addition, we analyzed the GO-based functional enrichment of genes in the AS network for BLCA, and a total of six GO terms were significantly enriched (**Figure 6C**). Ultimately, to evaluate the “performance” of these differential SFs, we performed a survival analysis and found that *NOVA1* was associated with survival-related ability, as were *ELAV4* and *ELAV3* (**Figure 7**).

### Prognosis-Associated Alternative Splicing Clusters

**Figure 8:**
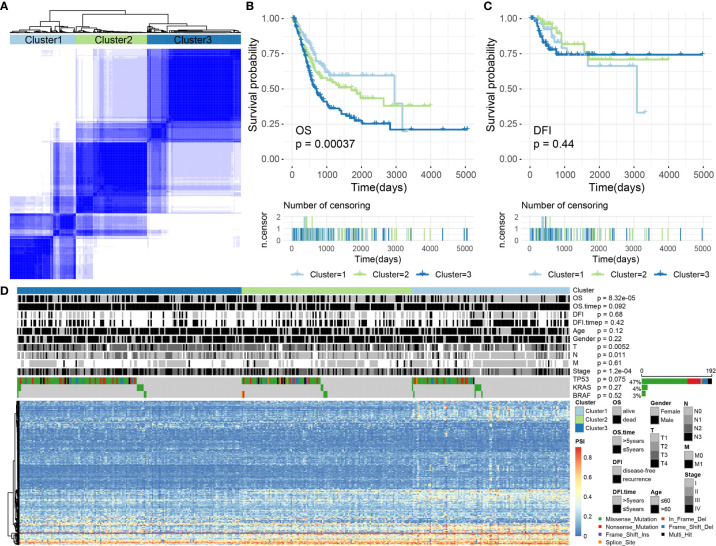
**(A)** The consensus matrix of BLCA defines three sample clusters. **(B, C)** Kaplan–Meier survival curves for BLCA associated with different AS clusters for OS and DFI. **(D)** Heatmap showing AS events and the distribution of clinical information across different AS clusters in BLCA.

We performed an unsupervised analysis of all selected samples based on the AS events in BLCA to further identify different AS patterns. According to a consensus cluster plus analysis, using a consensus value range from 0 (white, samples never gathered together) to 1 (dark blue, samples always gathered together), three groups of samples were categorized, as follows: C1 (n = 116, 28.57%), C2 (n = 125, 30.79%) and C3 (n = 165, 40.64%) (**Figure 8A**).

Subsequently, we conducted a survival analysis of BLCA samples to appraise the relevance of the identified clusters for OS/DFI prognosis. The results showed that AS clusters were associated with different OS survival modes (P = 0.0003680077, see **Figure 8B**) but not with different DFI survival modes (P = 0.4414947, see **Figure 8C**).

We further analyzed related information for BLCA samples, such as OS (alive or dead), DFI (disease-free or recurrence), survival time (OS/DFI > 5 years or ≤ 5 years), age (age > 60 or ≤ 60), sex (female or male), T, N, M, stage, and the presence of *TP53*, *KRAS*, *BRAF*, and other common cancer-driving genetic mutations. Some of this information was not randomly distributed. For example, discrepancies in the OS, T, N, and stage values were identified among the AS clusters associated with BLCA (Chi-square test, P-values < 0.05). Among these, the driving gene *TP53* was mutated in 192 samples (accounting for > 47%), but no significant difference was observed for the *TP53* distribution across the AS clusters (Chi-square test, P-values > 0.05; **Figure 8D**). Therefore, we were also able to identify molecular subtypes associated with prognoses through AS events.

### Sensitivity Differences to Immunotherapy and Chemotherapy Between the High- and Low-Risk Groups

**Figure 9:**
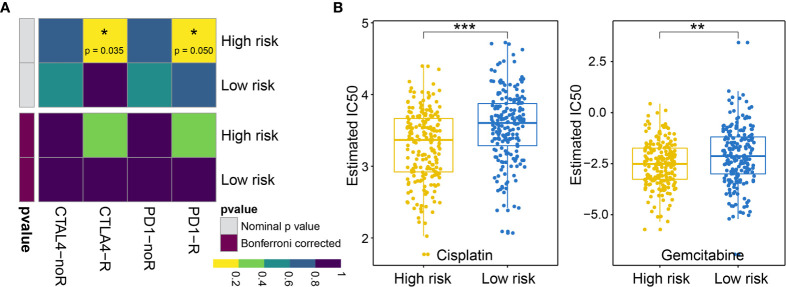
**(A)** Difference in the immunotherapy responses among BLCA patients at high and low risk of OS. **(B)** Differences in the cisplatin and gemcitabine chemotherapy responses among patients with BLCA at high and low risk of OS. * represents p values< 0.05, ** represents p values < 0.01, *** represents p values< 0.001.

First, we analyzed the response to immunotherapy in BLCA and used the TIDE algorithm to predict the response to immunotherapy. Notable differences in the responses to immunotherapy were observed between the high-risk group (19.10%, 38/199) and the low-risk group (57.58%, 114/198) (using Fishers exact test, p = 1.674e−15, and the Chi-square test, p = 7.065e−15). In addition to the TIDE prediction, we also compared the expression profiles of BLCA patients with high and low risk for OS using a submap algorithm, and we compared these outcomes with another data set derived from melanoma patients who were responsive to immunotherapy (17). We found that although no significant responses to immunotherapy were identified after correction *via* the Benjamini–Hochberg method in patients with high and low risk for OS, anti-programmed cell death protein 1 (PD-1) and cytotoxic T-lymphocyte-associated protein (CTLA4) therapy appears to be effective in the high-risk group without correction (PD-1 P = 0.04995005; CTLA4 P = 0.03496503; see **Figure 9A**).

Next, we considered the discrepancies in the responses to chemotherapy among BLCA patients and attempted to assess the differences in the responses to two chemicals (cisplatin and gemcitabine) between patients with high and low risk for OS. Thus, we trained a prediction model using the R package “pRRophetic” on the GDSC cell line dataset, using a ridge regression. We appraised its prediction accuracy through a ten-fold cross-validation. Based on the prediction model for these two chemicals, we estimated the IC_50_ values for each sample in the BLCA group. For these two chemicals, we observed significant differences in the IC_50_ values for cisplatin and gemcitabine in patients with high and low risk for OS associated with BLCA (cisplatin P = 1.918960e−07; gemcitabine P = 1.303591e−03; see **Figure 9B**).

## Discussion

Changes in AS events can have significant effects on oncogenesis and tumor progression (18). For example, the SF SF3B3 is upregulated and contributes to tumorigenesis by regulating *EZH2* pre-mRNA splicing, representing a key prognostic factor and therapeutic target in clear cell renal cell carcinoma (19). Similarly, many recent studies have shown that DASEs regulated by differentially expressed SFs have effects on tumorigenesis, the epithelial–mesenchymal transition, and lymphatic metastasis (12, 20–24). Therefore, analyses of DASEs can be meaningful in an oncogenic context. Alternative splicing is widely present in metazoans. The genes regulated by AS typically differ from DEGs, emphasizing a different biological process. **Figure 4** shows that DEGs can be differentially spliced, as can many non-DEGs, indicating that differential AS is a widespread regulatory mechanism that can act to supplement DEGs. We therefore aimed to emphasize the study of DASEs, rather than DEGs. To achieve this goal, we set the “percentage of samples with PSI value = 100%” and the average PSI > 0.05, which ensured that the incorporated DASEs occurred in all samples, making our analyses and models applicable to most cases. As for the gathering of DEGs, we used conventional methods with edge R package, and this can be regarded “another system” compared with the methods of gathering DASEs.

Given the potential importance of AS events in tumor biology, attention has been paid to the clinical relevance of AS events in cancer. Previous research based on TCGA datasets revealed the prognostic value of AS events in BLCA (3). Guo et al. reported that single-nucleotide polymorphisms can influence specific splicing events and are associated with BLCA risk scores (25). We also examined the profile and clinical relevance of AS events in BLCA by performing a pan-cancer analysis (26). Recently, some AS bioinformatics analyses reported the good performance of AS events in predicting prognosis (27–29). However, these studies have been based on SASEs, which explains their good prognosis-predicting performance. According to other studies (30, 31), the analysis of DASEs or cancer-specific AS events can also show significant results. In this study, we performed systematic analyses to determine the prognostic value of DASEs in BLCA. The results of univariate Cox regression analysis showed a strong correlation between DASEs and survival, suggesting that several DASEs events affect survival. The top 10 events identified in OS and DFI showed strong correlations with survival time. In the stepwise Cox regression analysis, independent prognostic AS events were identified in association with both OS and DFI. For the constructed prognostic model, however, the AUC value of OS was unfortunately not higher than 0.7, suggesting an insignificant result.

AS is regulated by a complex network and anomalous AS events and their associated regulatory factors should be investigated. Based on the differential AS genes, we constructed a PPI network to display how differential protein variants interact in BLCA (**Figure 4**). The plot offers a glimpse into changes in AS gene expression, AS types, and PPI. However, only a minority of the genes in the network have been identified as being alternatively spliced. For example, PKM exon 9 is skipped more frequently in BLCA (11). Although this network may be forward-looking, the available evidence to support the authenticity of this model is currently insufficient.

SFs are a series of RNA-binding proteins that can shear pre-RNA, and studying AS is vital. According to the network, eight SFs and numerous predictive pathways were associated with DASEs in BLCA. Little mechanism-based research exists for these eight SFs (CELF2, MBNL1, NOVA1, PTBP2, KHDRBS2, ELAVL2, ELAVL3, and ELAVL4) in BLCA; thus, further studies remain necessary. CELF2, an RNA-binding protein, can modulate RNA stability and translation by attaching to UG-rich sequence elements of introns, which can promote apoptosis and autophagy and regulate alternative polyadenylation (32–37). In this analysis, CELF2 was identified as a hub SF within the network; it was expressed at remarkably low levels and played a regulatory role for 37 DASEs. In addition, MBNL1 was the second most important SF. CELF2 and MBNL1 share some downstream genes and were both expressed at low levels, which agrees with the results of a recent research on the reciprocal regulatory roles of CELF2 and other SF (38). Most intensive studies have suggested that AS is regulated in a combinatorial manner by several SFs, which can be either synergistic or antagonistic (39). The cross-regulatory roles of SFs may have multifaceted effects for shaping cellular functions. Thus, further research referencing our AS network may be of great value. In the survival analysis, the SFs associated with DASEs did not present strong survival-related abilities. However, an increased potential population of downstream factors increases the functional complexity. These SFs were obtained by gap analysis, instead of survival analysis, which may explain why only NOVA1 appears to be a survival-related SF (**Figure 7**).

We did not identify any optimal prognostic AS clusters after conducting various classifications. BLCA has diverse biological specificity, suggesting that an increase in the number of clustering groups should be beneficial. According to the prognostic value of DASEs, we separated the sample into three groups of clusters related to prognoses in the case of OS while we failed to make the clustering relate to prognoses in the case of DFI. After overall consideration, we chose to retain this triple classification scheme.

AS events can also affect tumor immunity and sensitivity to chemotherapy drugs (40). To explore the immunotherapy response, the TIDE algorithm was used to determine significant differences in immunotherapy responses among the AS clusters (the responses were better in the low-risk group). Although the TIDE algorithm is the most effective method for predicting the immunotherapy response in melanoma (41), it may not be valid in other tumors. We have found that the TIDE algorithm appears to be useful for cervical squamous cell carcinoma (42) and BLCA. Predicting the response to immune checkpoint blockade therapy can be difficult, and only a small portion of patients obtain benefits from therapy; however, no currently available alternative methods can predict the response to immunotherapy. In this situation, any attempts to predict the immunotherapy response may be useful. We were able to identify differences in the immunotherapy response between groups according to OS. We then used a submap algorithm to predict whether differences could be identified in response to anti-PD-1 and anti-CTAL-4 between the low- and high-risk groups. Although no significant differences were detected after correction, the high-risk group showed promise for the response to anti-PD-1 and anti-CTAL-4 treatment without correction. In the prediction to chemotherapy response, cisplatin and gemcitabine showed significant differences between patients with high and low BLCA risks. We tested two clustering mechanisms, including AS clustering (dividing samples into three groups) and high/low-risk of OS grouping (mentioned in section 3.6), and found that risk grouping provided better predictive results.

Within this limited study, we systematically analyzed AS events, associated SFs, prognostic signatures, and sensitivity to immunotherapy and chemotherapy in BLCA. Further verification of these findings remains necessary through subsequent studies of DASEs and SFs, both *in vivo* and *in vitro*, and examining AS signatures in various population cohort studies is worth pursuing.

## Conclusion

Overall, we performed a novel study of the AS regulatory networks that may be involved in the oncogenesis of BLCA. In addition, an AS-based prognostic model was established, and the low-risk group showed greater sensitivity to immuno- and chemotherapy.

## Data Availability Statement

The datasets presented in this study can be found in online repositories. The names of the repository/repositories and accession number(s) can be found in the article/**Supplementary Material**.

## Author Contributions

CK, ZZ, and ZF designed the research study. ZF performed all the bioinformatics analyses described here. ZF wrote and edited the manuscript. CK and ZZ reviewed the article and made modification suggestions. CK and ZZ supervised the project. CP, ZL, and ZW offered advice. All authors contributed to the article and approved the submitted version.

## Funding

This work was supported by Shenyang Plan Project of Science and Technology (Grant No. F19-112-4-098), Natural Science Foundation of Liaoning Province (2019-MS-378) , Shenyang Clinical Medical Research Center (Grant No. 20-204-4-42).

## Conflict of Interest

The authors declare that the research was conducted in the absence of any commercial or financial relationships that could be construed as a potential conflict of interest.

## Abbreviations

AS, alternative splicing; BLCA, bladder urothelial carcinoma; CELF2, CUGBP Elav-Like Family Member 2; DASE, differential alternative splicing event; DFI, disease-free interval; MBNL1, muscleblind‐like 1**;** OS, overall survival.
