# Supplementary figures and images for: Genome-Wide Analyses of Prognostic and Therapeutic Alternative Splicing Signatures in Bladder Urothelial Carcinoma
Source: Front Oncol. 2021 Mar 26;11:626858. doi: 10.3389/fonc.2021.626858 (PMC8033158; doi:10.3389/fonc.2021.626858)

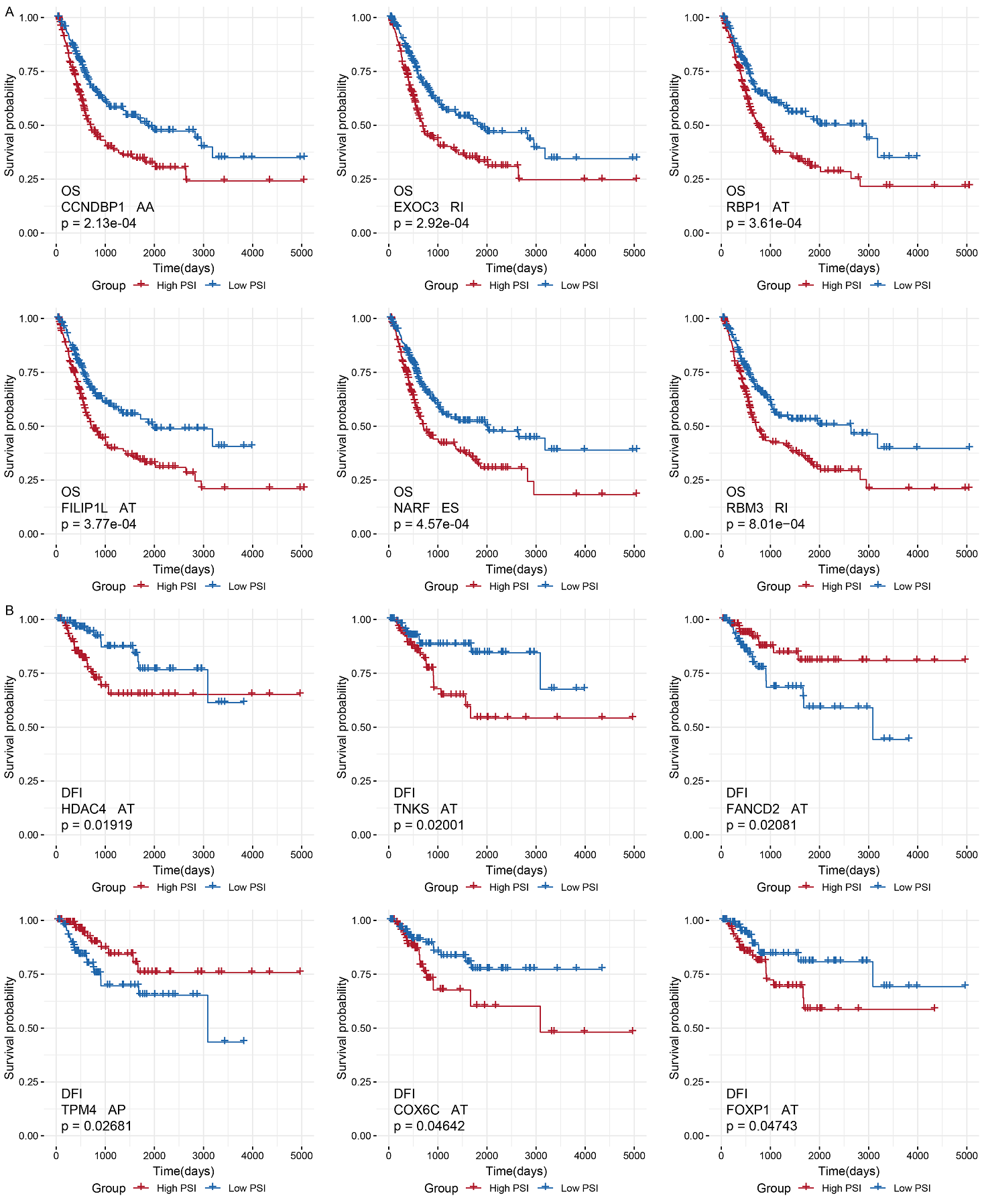

Supplement: Supplementary file 2 [file Image_1.tif]
